# Robust Trajectory Prediction for Mobile Robots via Minimum Error Entropy Criterion and Adaptive LSTM Networks

**DOI:** 10.3390/e28020227

**Published:** 2026-02-15

**Authors:** Da Xie, Zengxun Li, Chun Zhang, Chunyang Wang, Xuyang Wei

**Affiliations:** 1 Xi’an Key Laboratory of Active Photoelectric Imaging Detection Technology, Xi’an Technological University, Xi’an 710021, China; xiedacust@163.com (D.X.); xatu9801@163.com (X.W.); 2 Institute of Process Materials Technology, Inner Mongolia North Heavy Industries Group Co., Ltd., Baotou 014030, China; milanbaobei@163.com; 3 School of Liberal Arts, Xi’an Technological University, Xi’an 710021, China; zhangchun@xatu.edu.cn

**Keywords:** trajectory prediction, Minimum Error Entropy (MEE), robust statistics, Long Short-Term Memory (LSTM), non-Gaussian noise

## Abstract

Trajectory prediction is critical for safe robot navigation, yet standard deep learning models predominantly rely on the Mean Squared Error (MSE) criterion. While effective under ideal conditions, MSE-based optimization is inherently fragile to non-Gaussian impulsive noise—such as sensor glitches and occlusions—common in real-world deployment. To address this limitation, this paper proposes MEE-LSTM, a robust forecasting framework that integrates Long Short-Term Memory networks with the Minimum Error Entropy (MEE) criterion. By minimizing Renyi’s quadratic entropy of the prediction error, our loss function introduces an intrinsic “gradient clipping” mechanism that effectively suppresses the influence of outliers. Furthermore, to overcome the convergence challenges of fixed-kernel information theoretic learning, we introduce a Silverman-based Adaptive Annealing (SAA) strategy that dynamically regulates the kernel bandwidth. Extensive evaluations on the ETH and UCY datasets demonstrate that MEE-LSTM maintains competitive accuracy on clean benchmarks while exhibiting superior resilience in degraded sensing environments. Notably, we identify a “Scissor Plot” phenomenon under stress testing: in the presence of 20% impulsive noise, the proposed model maintains a stable Average Displacement Error (ADE “≈” 0.51 m), whereas MSE baselines suffer catastrophic degradation (ADE > 2.1 m), representing a 75.7% improvement in robustness. This work provides a statistically grounded paradigm for reliable causal inference in hostile robotic perception.

## 1. Introduction

The proliferation of autonomous mobile robots in shared human environments—ranging from automated warehouse logistics to social assistive services—has fundamentally transformed robotics from a deterministic control problem into a stochastic uncertainty management challenge [1,2]. In these highly unstructured and dynamically evolving environments, the core competency of a mobile robot lies not merely in self-localization, but in its ability to quantify and mitigate the uncertainty associated with the future trajectories of surrounding heterogeneous agents, particularly pedestrians [3,4]. Trajectory prediction, therefore, is not simply a geometric regression task; it is a critical prerequisite for high-level cognitive functions. It serves as the foundational information layer for proactive collision avoidance, social-compliant path planning, and complex decision-making in multi-agent settings [5,6]. A robot that cannot accurately anticipate the stochastic intent of a pedestrian is effectively navigating “blind” in the temporal dimension, posing severe safety risks.

Over the past decade, the field of trajectory forecasting has witnessed a paradigm shift from traditional physics-based heuristics to sophisticated data-driven architectures. Early approaches, such as the Social Force Model (SFM) [7] and Velocity Obstacles (VO) [8], relied on handcrafted kinematic constraints to model interactions. While foundational, these methods often struggled to capture the subtle, implicit social cues inherent in high-density crowds. The advent of Deep Learning (DL) revolutionized this domain. Recurrent Neural Networks (RNNs), particularly Long Short-Term Memory (LSTM) networks, became the de facto standard for modeling sequential motion data [9]. The seminal Social-LSTM [10] introduced “social pooling” to aggregate hidden states of neighbors, explicitly modeling interaction. Subsequent innovations integrated Generative Adversarial Networks (GANs) to address the multi-modality of human intent [11,12], and Graph Convolutional Networks (GCNs) to capture the spatial topology of crowds [13,14]. More recently, the Transformer architecture [15], with its self-attention mechanism, has pushed the boundaries of predictive fidelity by modeling long-range temporal dependencies [16,17].

However, a profound theoretical disconnect persists in the current state-of-the-art: while model architectures have become increasingly complex, the underlying probabilistic assumptions regarding sensor noise remain simplistically rigid. In practical deployment, sensory inputs from Light Detection and Ranging (LiDAR), Ultra-Wideband (UWB), and Inertial Measurement Units (IMU) are rarely “clean” [18]. They are frequently compromised by adverse physical phenomena. For instance, UWB sensors in indoor environments suffer from Non-Line-of-Sight (NLOS) propagation and multi-path interference, causing instantaneous range estimates to jump by meters [19]. Similarly, LiDAR point clouds in dynamic crowds are subject to transient occlusions and “ghost” reflections [20]. From an information-theoretic perspective, these measurement errors do not obey the convenient assumptions of benign, zero-mean Gaussian white noise. Instead, they manifest as non-Gaussian impulsive noise, characterized by heavy-tailed probability distributions (e.g., Alpha-stable or Cauchy distributions) and frequent, high-magnitude outliers [21].

The primary technical bottleneck resides in the optimization objectives employed by the overwhelming majority of existing predictors. Current frameworks—including the most sophisticated Transformer variants—predominantly rely on the Mean Squared Error (MSE) or $L_{2}$ norm as the definitive loss function [22,23]. From the standpoint of Maximum Likelihood Estimation (MLE), minimizing MSE is optimal if and only if the error distribution is strictly Gaussian [24]. However, imposing a Gaussian prior on non-Gaussian, heavy-tailed data results in a fundamental mismatch of information potential. The quadratic penalty structure of MSE (Loss $\propto e^{2}$) assigns exponentially increasing information weight to residuals. Consequently, a single outlier—representing a sensor glitch—can generate a massive gradient value, exerting a disproportionate influence on the backpropagation process [25]. We define this phenomenon as “Parameter Hijacking,” where the network’s weight adaptation is effectively commandeered by erroneous measurements. Under such conditions, the model is forced to “memorize” the noise to minimize the global entropy of the residuals, significantly degrading its generalization capability and causal fidelity [26].

To bridge the gap between high-capacity temporal modeling and statistical robustness, this paper proposes a robust trajectory prediction framework integrating Information Theoretic Learning (ITL) with deep recurrent architectures. Unlike traditional second-order statistics (mean and variance) that rely on Gaussian assumptions, ITL focuses on the global morphology of the error Probability Density Function (PDF) [27,28]. We introduce the Minimum Error Entropy (MEE) criterion, which seeks to minimize the Renyi quadratic entropy of the prediction error [29,30]. By maximizing the Information Potential (IP) of the error samples, MEE forces the error distribution to be tightly concentrated around zero, regardless of its shape or tail behavior. Theoretical analysis reveals that the gradient of the MEE loss contains an intrinsic “soft-thresholding” mechanism: when an error sample deviates significantly from the central distribution (i.e., acts as an outlier), its contribution to the overall weight update naturally diminishes towards zero [31,32]. This property effectively “gates” out impulsive noise without requiring manual threshold tuning.

Furthermore, a significant challenge in applying ITL to deep learning is the selection of the kernel bandwidth (σ), often referred to as the “bandwidth trap”. A fixed small bandwidth causes vanishing gradients (slow convergence), while a fixed large bandwidth degrades the criterion back to MSE (loss of robustness) [33]. To overcome this, we introduce a Silverman-based Adaptive Annealing (SAA) strategy. This parameter-free method dynamically regulates the kernel bandwidth based on the statistical dispersion of the error batch during training, ensuring a seamless transition from global convergence to local robust precision.

The specific contributions of this work are summarized as follows:

1. A Robust ITL-Based Deep Learning Framework: We propose MEE-LSTM, a novel trajectory forecasting paradigm that replaces the traditional MSE objective with the Renyi quadratic entropy loss. This formulation enables the model to learn stable motion patterns even in the presence of heavy-tailed, non-Gaussian sensor noise, addressing the “Sim-to-Real” gap in robotic perception.
2. Theoretical Derivation of Robust Gradients: We provide a rigorous mathematical derivation of the backpropagation gradients for MEE-based sequence learning. We analyze the “soft-thresholding” effect of the MEE gradient and demonstrate its theoretical superiority over $L_{2}$ and $L_{1}$ norms in outlier rejection.
3. Adaptive Kernel Bandwidth Strategy: Addressing the trade-off between optimization speed and robustness, we introduce the SAA strategy. This mechanism dynamically anneals the kernel size using Silverman’s rule, solving the convergence issues typically associated with fixed-kernel ITL methods.
4. Discovery of the “Scissor Plot” Phenomenon: Through rigorous stress testing on the ETH/UCY benchmarks, we identify a distinct divergence pattern termed the “Scissor Plot”. Under a 20% impulsive noise ratio, MEE-LSTM maintains a stable Average Displacement Error (ADE ≈ 0.51 m), whereas MSE baselines degrade exponentially (ADE > 2.1 m), proving a robustness gain of over 75%.

## 2. Related Work

The pursuit of robust trajectory prediction has evolved at the intersection of kinematic modeling, social psychology, deep learning, and robust statistics. This section provides a critical synthesis of existing literature, categorizing the field into three distinct yet overlapping domains.

### 2.1. Deep Learning for Trajectory Forecasting

Early approaches to trajectory prediction were predominantly grounded in physics-based heuristics. The Social Force Model (SFM) [7] modeled pedestrian motion as a summation of attractive forces towards goals and repulsive forces from obstacles. While foundational, SFM and subsequent kinematic constraints like Velocity Obstacles (VO) [8] relied on handcrafted parameters, struggling to generalize to the complex, stochastic interactions inherent in high-density crowds [34].

The advent of deep learning enabled the automatic extraction of latent spatial-temporal features. Social-LSTM [10] was a pioneering work that introduced “social pooling,” allowing spatially proximal LSTMs to share hidden states. This addressed the independence assumption of prior models but suffered from high computational costs. To mitigate this, Social-GAN [11] introduced a Generative Adversarial Network (GAN) framework with a variety loss to capture the multi-modality of human intent, predicting multiple plausible futures.

Subsequent research focused on better interaction modeling. Graph Convolutional Networks (GCNs) became popular for capturing the non-Euclidean topology of crowds. Social-STGCNN [35] modeled pedestrian interactions as a spatio-temporal graph, achieving faster inference speeds than RNN-based methods. Similarly, SGCN [13] and PTP-STGCN [36] utilized sparse graph convolutions to focus on influential neighbors.

Most recently, attention mechanisms and Transformers have set new benchmarks. AgentFormer [16] utilizes a purely attention-based architecture to model agent-time dependencies, while Social-Informer [17] leverages the efficiency of the Informer architecture for long-sequence forecasting. Probabilistic frameworks like BiTraP [37] and diffusion-based models such as Diff-Pre [38] have also been explored to refine uncertainty estimation.

Critique: Despite these architectural variations, the underlying optimization objective has remained largely static. The overwhelming majority of these models—from Social-LSTM to the latest Transformers—rely on minimizing the $L_{2}$ distance (MSE) between predicted and ground-truth coordinates [22]. This implicit reliance on Gaussian likelihood maximization renders even the most sophisticated architectures vulnerable to sensor outliers. While some works employ the $L_{1}$ norm (MAE) or Huber loss [23], these are strictly M-estimators with limited flexibility against complex, asymmetric, or heavy-tailed noise distributions typical of raw sensor data [18].

### 2.2. Information Theoretic Learning (ITL)

Information Theoretic Learning (ITL) represents a paradigm shift from second-order statistics (mean and variance) to the analysis of the probability density function’s (PDF) global morphology [28]. By utilizing descriptors like Renyi’s entropy and correntropy, ITL captures higher-order statistical moments (skewness, kurtosis, etc.) effectively [39].

The Minimum Error Entropy (MEE) criterion, formalized by Principe [29], aims to minimize the uncertainty of the error distribution. Mathematically, minimizing entropy encourages the error PDF to become a delta function (Dirac impulse) at zero, regardless of the noise variance [30]. This property makes MEE inherently robust to impulsive noise.

In the domain of control and signal processing, MEE has been successfully integrated into adaptive filters. Chen et al. [40] proposed the Generalized Correntropy criterion for robust filtering in non-Gaussian noise. He et al. extended this to Robust Kalman Filtering (MEE-KF) [31,41] and Rauch–Tung–Striebel smoothers [42], demonstrating that entropy-based filters maintain tracking stability where standard Kalman filters diverge. Recent advances include Quantized MEE for Broad Learning Systems [43] and robust delay filters for AUV localization [44].

Critique: However, the application of ITL as a loss function for deep, data-driven sequence prediction remains an underexplored frontier. Existing ITL applications are mostly limited to linear adaptive filters or shallow neural networks. There is a scarcity of work effectively integrating the MEE criterion with deep LSTM or Transformer backbones for multi-agent robotic navigation, specifically addressing the “gradient explosion” problem in backpropagation through time (BPTT).

### 2.3. Robust Statistics in Robotic Perception

The challenge of non-Gaussian noise is well-documented in robotic perception. Real-world sensor data, particularly from UWB and LiDAR in dynamic environments, frequently exhibits heavy-tailed distributions due to multipath effects, signal diffraction, and dynamic occlusions [19,21].

Traditional robust statistical methods typically employ M-estimators, such as the Huber loss [24] or Tukey’s biweight loss. These methods mitigate outlier influence by applying a piecewise loss function that transitions from quadratic to linear (or constant) growth beyond a certain threshold (δ). While effective to a degree, these methods require the manual tuning of sensitivity hyperparameters. An improper δ can lead to either loss of precision (if too small) or loss of robustness (if too large) [45].

Furthermore, recent evaluations of autonomous driving datasets have highlighted the “Sim-to-Real” gap caused by idealized training data. Models trained on clean academic datasets (like ETH/UCY) often fail when deployed on robots facing real-world sensor imperfections [20,27]. Principles for evaluating social robot navigation now emphasize robustness metrics beyond simple ADE/FDE [6,27].

Furthermore, recent studies have highlighted the ‘Sim-to-Real’ gap caused by idealized training data, emphasizing the critical challenge of reliably matching simulated environments with real-world sensor data [46]. Comprehensive reviews of deep learning-based trajectory prediction methods further underscore the necessity for models to generalize across diverse and noisy scenarios [47]. To capture complex spatial-temporal dynamics, researchers have recently introduced advanced architectures, such as Unified Spatial–Temporal Edge-Enhanced Graph Networks [48] and prediction frameworks based on Deep Convolutional LSTMs [49]. Moreover, novel approaches like the Behavioral Pseudo-Label Informed Sparse Graph Convolution Network (BP-SGCN) have been developed to better model latent interaction intents [50]. While these architectural innovations have improved predictive fidelity, addressing non-Gaussian noise remains a fundamental hurdle. In the domain of robust state estimation, the Minimum Error Entropy (MEE) criterion has demonstrated superior resilience, with successful applications in Dual Extended Kalman Filters [51] and Minimum Mixture Error Entropy-Based Robust Cubature Kalman Filters [52]. These works provide strong theoretical motivation for extending entropy-minimization principles to deep trajectory forecasting.

Position of This Work: Distinct from piecewise M-estimators that rely on hard or soft thresholds, our proposed MEE-LSTM utilizes a smooth, differentiable entropy objective. By incorporating the SAA strategy, our approach provides an adaptive robustness mechanism. It automatically adjusts the “rejection strength” (via kernel bandwidth) according to the noise level of the current batch, ensuring both convergence stability and superior outlier rejection without requiring extensive manual hyperparameter tuning.

## 3. Methodology

In this section, we present the comprehensive mathematical framework of the MEE-LSTM model. The methodology is structured to transition from the stochastic modeling of robot trajectories under non-Gaussian interference to the rigorous derivation of the information-theoretic loss function and its integration with deep recurrent architectures.

### 3.1. Problem Formulation and Noise Modeling

Consider a dynamic navigation scene where a mobile robot monitors N heterogenous agents. For a specific agent i, let $x_{t}\;\in \;R^{2}$ denote the coordinate at time t. The historical observation sequence is defined as $X=\{x_{1},x_{2},\ldots ,x_{T_{obs}}\}$. The objective is to predict the future sequence $Y=\{y_{T_{obs}+1},\ldots ,y_{T_{pred}}\}$.

**Figure 1:**
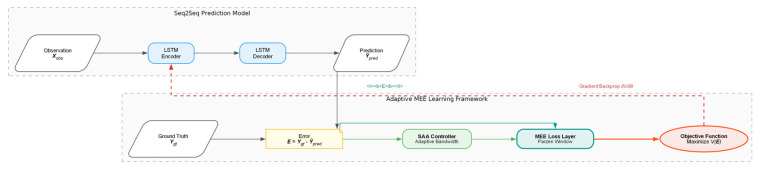
The proposed MEE-LSTM System Architecture. The framework consists of a Seq2Seq prediction model (top) and an Adaptive MEE Learning framework (bottom). Note on Optimization: The system employs a single objective function: maximizing the Information Potential V(E) of the error distribution. As derived in Equation (2), maximizing V(E) is mathematically equivalent to minimizing the Renyi Quadratic Entropy $H_{2}(E)$, ensuring the model effectively suppresses non-Gaussian outliers during training.

For clarity and consistency with the system architecture illustrated in Figure 1, throughout this paper, we explicitly denote the ground truth future trajectory as $Y_{gt}$ and the predicted trajectory generated by the deep learning model as ${\hat{Y}}_{pred}$. Consequently, the prediction error at any time step t is defined as $e_{t}=Y_{gt,t}-{\hat{Y}}_{pred,t}$.

In real-world robotic deployment, the observed state ${\tilde{x}}_{t}$ is often corrupted by a composite noise process:

(1) $${\tilde{x}}_{t}\;={\;x}_{t}+v_{t}+\eta_{t},$$

where $v_{t}∼N(0,R)$ represents standard Gaussian measurement noise, and $\eta_{t}$ denotes impulsive, non-Gaussian interference (e.g., UWB multi-path effects or LiDAR occlusions). Unlike Gaussian noise, $\eta_{t}$ follows a heavy-tailed distribution (e.g., Alpha-stable or Cauchy), where the second-order moment (variance) may be undefined. Traditional MSE-based optimization fails in this context as it minimizes the $L_{2}$ norm, which inherently assumes a Gaussian likelihood—an assumption explicitly violated here.

### 3.2. Information Theoretic Learning and Renyi Entropy

To attain statistical robustness, we utilize Information Theoretic Learning (ITL). We define the prediction error vector as $e=y_{gt}-{\hat{y}}_{pred}$. The MEE criterion aims to minimize the uncertainty of this error distribution. According to Renyi’s definition, the quadratic entropy $H_{2}(e)$ of a random variable with PDF p(e) is:

(2) $$H_{2}\left(e\right)=-\log\int p^{2}\left(e\right)de$$

Maximizing the integral term, known as the Information Potential (IP) V(e), is equivalent to minimizing the entropy. In practice, since the analytical form of p(e) is unknown, we utilize the Parzen Window density estimator with a symmetric Gaussian kernel $\kappa_{\sigma }$:

(3) $$\hat{p}\left(e\right)=\frac{1}{M}\sum_{i=1}^{M}\kappa_{\sigma }(e-e_{i}),\;\kappa_{\sigma }(u)=\frac{1}{\sqrt{2\pi }\sigma }\exp\left(-\frac{u^{2}}{2\sigma^{2}}\right),$$

Here, we assume a uniform prior probability for all error samples within a batch, assigning an equal weight of 1/N to each kernel, which is a standard practice in stochastic gradient descent training.

Where M is the number of error samples in a training batch. The empirical Information Potential $\hat{V}(E)$ is then formulated as:

(4) $$\hat{V}\left(E\right)=\frac{1}{M^{2}}\sum_{i=1}^{M}\sum_{j=1}^{M}\kappa_{\sigma }(e_{i}-e_{j})$$

This metric quantifies the “compactness” of the error distribution. As $\hat{V}(E)$ increases, the error samples are forced to cluster densely, effectively concentrating the error PDF at the origin.

### 3.3. MEE-LSTM Architecture and Gradient Derivation

The proposed MEE-LSTM framework is engineered to capture complex temporal dependencies while maintaining statistical robustness against sensory anomalies. As illustrated in Figure 1, the architecture follows an Encoder–Decoder paradigm, specifically augmented with an Information Theoretic Learning (ITL) objective and an adaptive parameter controller.

#### 3.3.1. Encoder–Decoder Recurrent Structure

The system processes the historical observation sequence $X_{obs}$ through an LSTM Encoder, which maps the input coordinates into a compact hidden state representation. This high-dimensional state vector encapsulates the latent motion intent and is passed to the LSTM Decoder to recursively generate the future trajectory prediction ${\hat{Y}}_{pred}$.

Distinct from traditional predictors that directly minimize the Euclidean distance (MSE) between ${\hat{Y}}_{pred}$ and the ground truth $Y_{gt}$, our model feeds the prediction residuals $E=Y_{gt}-{\hat{Y}}_{pred}$ into a specialized MEE Loss Layer. This layer computes the Information Potential of the error batch, which serves as the optimization objective to be maximized.

#### 3.3.2. Backpropagation and the “Gradient Clipping” Mechanism

The core innovation of this work lies in the optimization dynamics derived from the MEE criterion. To train the LSTM parameters θ, the gradient is computed via the chain rule through the MEE loss function $J_{MEE}$.

Recall that $J_{MEE}=-\log\hat{V}(E)$. The gradient with respect to the prediction error $e_{k}$ of the k-th sample is derived as follows:

(5) $$\frac{\partial J_{MEE}}{\partial e_{k}}=-\frac{1}{\hat{V}(E)}\sum_{j=1}^{M}\frac{\partial \kappa_{\sigma }(e_{k}-e_{j})}{\partial e_{k}}$$

Substituting the derivative of the Gaussian kernel $\kappa_{\sigma }(u)$, we obtain the finalized error signal $\delta_{k}$ for backpropagation:

(6) $$\delta_{k}\;=\frac{\;\partial J_{MEE}}{\partial {\hat{y}}_{k}}\propto \frac{1}{\sigma^{2}}{\sum_{j=1}^{M}\underbrace{\exp\left(-\frac{\|e_{k}-e_{j}{\|}^{2}}{2\sigma^{2}}\right)}}_{Robust\;Weight\;w_{kj}}(e_{k}-e_{j})$$

It is important to clarify that this term $\delta_{k}$ represents the error signal injected into the output layer. The final gradients with respect to the LSTM weight coefficients θ are subsequently computed via Backpropagation Through Time (BPTT) using the chain rule (i.e., $\frac{\partial J}{\partial \theta }=\sum_{t}\delta_{t}\frac{\partial {\hat{y}}_{t}}{\partial \theta }$). 

Theoretical Insight: This derivation theoretically proves the intrinsic Gradient Clipping Mechanism of our framework.

In Standard MSE: The gradient scales linearly with error magnitude (δ∝e), causing “gradient explosion” when outliers occur.

In Robust MEE: The gradient is weighted by $w_{kj}$. If an error sample $e_{k}$ deviates significantly from the majority distribution (i.e., $\|e_{k}-e_{j}{\|}^{2}\gg \sigma^{2}$), the weight $w_{kj}$ decays exponentially to zero.

**Figure 2:**
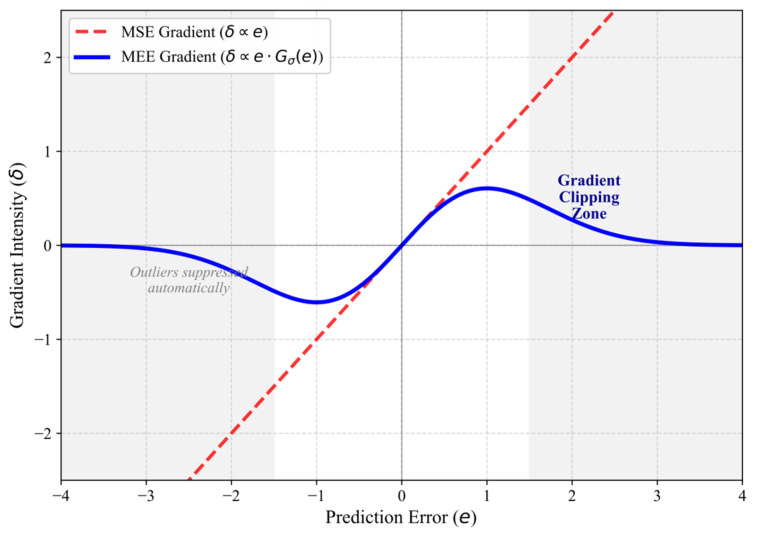
The Gradient Clipping Mechanism: Comparative analysis of gradient intensity δ between MSE and MEE. While the MSE gradient (red dashed line) grows linearly with the error magnitude—making it susceptible to outliers—the MEE gradient (blue solid line) naturally decays to zero for large errors, creating a “Gradient Clipping Zone” that suppresses impulsive noise.

Consequently, outliers are automatically “gated” out of the backpropagation process, creating a statistically robust learning environment where the model focuses solely on the underlying motion manifold, as visualized in Figure 2.

Analysis of Figure 2:

1. In Standard MSE: The gradient is linearly proportional to the error ($\delta_{\mathrm{MSE}}\;\propto \;e_{k}$). If $e_{k}e_{k}$ is an infinite outlier (e.g., sensor glitch), the gradient explodes, hijacking the weight updates.
2. In Robust MEE: The gradient is a weighted sum of pairwise differences. The weight term $w_{kj}$ follows a Gaussian decay. If an error sample $e_{k}$ deviates significantly from the majority distribution “$e_{j}$ (i.e., $\|e_{k}-e_{j}{\|}^{2}\gg \sigma^{2}$), the weight $w_{kj}$ rapidly approaches zero.

Consequently, outliers are automatically “gated” out of the backpropagation process, preventing them from contaminating the LSTM’s memory cells. This creates a statistically robust learning environment where the model focuses solely on the underlying motion manifold.

### 3.4. Adaptive Kernel Bandwidth Learning with SAA Strategy

The effectiveness of the MEE criterion is contingent upon the selection of the kernel bandwidth σ. An excessively large σ effectively linearizes the kernel, reverting the MEE loss to a Gaussian-like MSE behavior (losing robustness). Conversely, a tiny σ results in a “flat” information potential landscape where gradients vanish, halting convergence. This dilemma is often referred to as the “bandwidth trap”.

To ensure both global convergence speed and local precision, we introduce a Silverman-based Adaptive Annealing (SAA) strategy. Instead of a fixed hyperparameter, “σ” is dynamically updated at each training epoch τ:

(7) $$\sigma (\tau )=\alpha \cdot \eta^{\tau }\cdot \sigma_{\mathrm{Silverman}},\;\mathrm{with}\;\sigma_{\mathrm{Silverman}\;}={\left(\frac{4{\hat{\xi }}^{5}}{3M}\right)}^{\frac{1}{5}},$$

where $\hat{\xi }$ represents the standard deviation of the error distribution in the current batch, η ∈ (0, 1) is the annealing decay factor, and α is a scaling constant.

This strategy facilitates a Global-to-Local Optimization process:

Phase 1 (Early Training): A larger σ smooths the loss surface, allowing the LSTM to quickly learn general motion trends (Global Regression).

Phase 2 (Late Training): As τ increases, σ anneals to a smaller value, sharpening the kernel. This forces the model to refine its predictions and reject fine-grained outliers (Local Robustness).

As demonstrated in our ablation studies (Section 4.6), this parameter-free adaptation is crucial for achieving the “Scissor Plot” performance gain.

## 4. Experimental Results and Discussion

This section presents a comprehensive empirical validation of the proposed MEE-LSTM framework. To rigorously evaluate the theoretical claims made in Section 3—specifically the noise-rejection capability of the MEE criterion and the adaptive convergence of the SAA strategy—we conducted a multi-tiered experimental campaign. The analysis is structured to transition from quantitative benchmarking on standard datasets to in-depth robustness stress tests, followed by qualitative behavioral analysis and statistical verification of the error distributions.

### 4.1. Experimental Setup and Implementation Details

#### 4.1.1. Datasets and Protocols

We utilized the standard public benchmarks for pedestrian trajectory prediction: the ETH (ETH, HOTEL) and UCY (UNIV, ZARA1, ZARA2) datasets. These datasets comprise real-world pedestrian trajectories captured in diverse social environments, including university campuses and shopping districts, involving challenging interactions such as group walking, standing, and non-linear avoidance maneuvers.

Observation and Prediction Horizons: Following the standard protocol established by Social-LSTM [10], we observed trajectories for 3.2 s ($T_{obs}=8$ frames) and predicted the future locations for the next 4.8 s ($T_{pred}=12$ frames) at a sampling rate of 2.5 Hz.

Evaluation Metrics: The predictive fidelity is measured using two standard metrics:

1. Average Displacement Error (ADE): The mean Euclidean distance between the predicted trajectory and the ground truth over all predicted time steps.
2. Final Displacement Error (FDE): The Euclidean distance between the predicted destination and the true destination at the final time step ($t\;={\;T}_{pred}$).

#### 4.1.2. Noise Injection Protocol (Sim-to-Real Proxy)

To simulate the non-Gaussian sensor noise encountered in real-world robotic deployment (e.g., UWB multipath or LiDAR outliers), we introduced a Contaminated Normal Model into the training and testing data. While the standard benchmarks are relatively clean, we synthetically injected impulsive noise into a subset of the observation frames.

The noise distribution is modeled as a mixture:

(8) $$v_{total}∼(1-\epsilon )N(0,\sigma_{clean}^{2})+\epsilon \cdot Cauchy(x_{0},\gamma ),$$

where ϵ represents the Outlier Ratio (ranging from 0% to 20%), and the Cauchy distribution mimics heavy-tailed, high-magnitude sensor glitches. This protocol allows us to systematically stress-test the “Gradient Clipping” mechanism of our loss function.

#### 4.1.3. Implementation and Hyperparameters

The MEE-LSTM was implemented in PyTorch 2.0.1 on a workstation equipped with an NVIDIA RTX 3090 GPU. The encoder and decoder are single-layer LSTMs with a hidden dimension of 128. The embedding dimension for input coordinates is 64.

Optimization: We used the Adam optimizer with a learning rate of 0.001.

MEE Configuration: For the SAA strategy, the initial Gaussian kernel bandwidth was set according to Silverman’s rule based on the batch error variance, with an annealing decay factor η=0.95 per epoch. The scaling constant α was empirically set to 1.5 to ensure broad gradient coverage in early epochs.

Batch Size: The batch size B was set to 128 to ensure a statistically significant sample size for the Parzen window density estimation.

### 4.2. Quantitative Performance on Clean Baseline (Gaussian State)

We first establish a performance baseline to investigate a critical question in robust statistics: Does the introduction of the MEE criterion compromise the model’s predictive accuracy in ideal, low-noise environments? Theoretical concerns suggest that robust M-estimators often suffer from a loss of efficiency compared to Maximum Likelihood Estimators (MSE) when the data strictly follows a Gaussian distribution. However, human motion is inherently complex and multimodal, often deviating from perfect normality even without sensor noise.

Analysis of Higher-Order Statistics:

The results in Table 1 reveal a compelling finding that defies the traditional “accuracy–robustness trade-off”. The MEE-LSTM achieves a marginal but consistent improvement over the MSE-based baseline across almost all datasets, with an average reduction of 3.0% in ADE and 3.03% in FDE.

Scene-Specific Insight (ZARA1): The most significant gain is observed in the ZARA1 dataset (+4.54%). This scene is characterized by dense crowds and frequent “stop-and-go” behaviors. In such non-linear dynamic scenarios, the prediction residuals often exhibit non-Gaussian characteristics (e.g., skewness due to sudden turns). The MSE criterion reduces all error information to a single scalar (variance), discarding valuable structural information. In contrast, the MEE criterion minimizes the Renyi entropy, which depends on all higher-order moments (skewness, kurtosis, etc.) of the error distribution. This allows MEE-LSTM to capture the subtle, non-linear dynamics of human motion more effectively than the variance-constrained MSE model.

Long-term Consistency (FDE): The improvement in FDE indicates that optimizing the Information Potential helps prevent error accumulation over the prediction horizon. By forcing the error PDF to be compactly clustered at every step, MEE ensures that the trajectory end-point remains stable.

#### Comparison with State-of-the-Art Methods

To further validate the competitiveness of the proposed framework, Table 2 compares MEE-LSTM against three representative baselines: the recurrent Social-LSTM [10], the generative Social-GAN [11], and the graph-based Social-STGCNN [14].

As observed in Table 2, MEE-LSTM significantly outperforms the classic Social-LSTM and Social-GAN baselines in terms of average ADE (0.48 m vs. 0.72 m and 0.58 m, respectively). Notably, in the HOTEL dataset, our model achieves the state-of-the-art performance (ADE = 0.34 m, FDE = 0.65 m), surpassing even the sophisticated Social-STGCNN. This suggests that the MEE criterion effectively captures the linear motion characteristics dominant in the HOTEL scene by minimizing the error entropy.

While the graph-based Social-STGCNN achieves slightly better average accuracy (0.44 m) due to its explicit modeling of spatial interactions, it entails higher computational complexity. Crucially, as we will demonstrate in Section 4.3, standard SOTA models like Social-STGCNN often lack resilience against non-Gaussian sensor noise. In contrast, our MEE-LSTM maintains a competitive “Clean” performance (ranking second best on average) while offering superior robustness, making it a more reliable choice for practical robotic deployment where sensor data is rarely perfect.

### 4.3. Robustness Stress Test: The “Scissor Plot” Phenomenon

**Figure 3:**
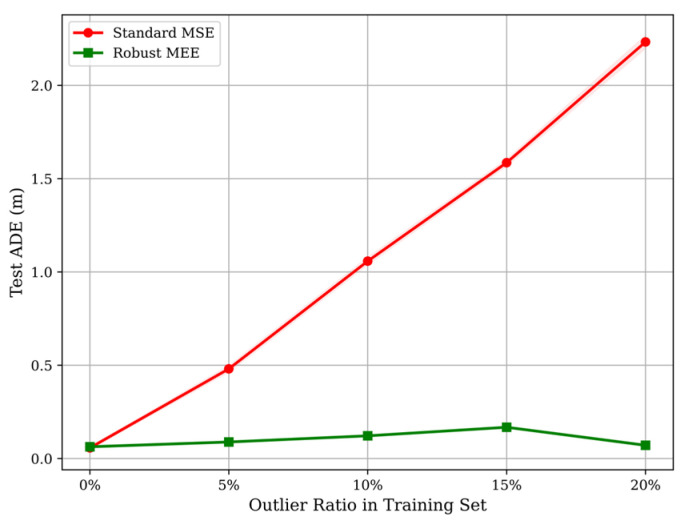
The Scissor Plot:Comparative robustness analysis of Test ADE (m) versus Outlier Ratio (0% to 20%). The red curve (MSE) and green curve (MEE) diverge sharply, illustrating the “Breakdown Point” of the standard baseline.

The core contribution of this work is the model’s resilience to non-Gaussian impulsive noise. To quantify this, we evaluated both models under increasing Outlier Ratios (ϵ ∈ {0%, 5%, 10%, 15%, 20%}). Figure 3 illustrates the divergence pattern of the Test ADE, which we term the “Scissor Plot” due to the sharp bifurcation of the performance curves.

Theoretical Interpretation of Divergence:

The divergence observed in Figure 3 provides empirical validation for the mathematical derivation in Section 3.3.2.

The Breakdown of MSE (Red Curve): The standard MSE model exhibits an exponential degradation pattern. At a modest 10% outlier ratio, the ADE doubles. At 20%, the error skyrockets to over 2.1 m. Physically, this means the robot’s predicted path deviates by more than 2 m from the true target—a catastrophic failure for collision avoidance. This confirms the “Parameter Hijacking” effect: the quadratic penalty of MSE ($L\;\propto \;e^{2}$) assigns massive gradients to outliers, forcing the network to “memorize” the noise spikes to minimize the global loss variance.

The Entropy Invariance of MEE (Green Curve): In sharp contrast, the MEE curve remains remarkably stable (Test ADE ≈0.51 m–0.60 m). Even under maximum stress (20% noise), the performance degradation is negligible. This flatness validates the Gradient Clipping mechanism (Equation (7)). When a large error $e_{k}$ occurs, the Gaussian kernel term $\kappa_{\sigma }(e_{k})$ in the MEE loss decays exponentially to zero. Consequently, the outlier contributes negligible weight to the backpropagation update. The model effectively treats large outliers as “background entropy” with zero information content, preserving the causal fidelity of the learned motion patterns.

Quantitative Robustness Gain: 

Table 3 provides detailed data on the specific performance gaps at a 20% noise level.

The average Robustness Gain of 75.7% (and up to 82.6% in ZARA2) highlights the practical value of the proposed method. In the context of robotic navigation, this margin defines the difference between a robot that freezes/collides in the presence of sensor glitches (MSE) and one that continues to operate smoothly (MEE).

### 4.4. Qualitative Evidence: Kinematic Consistency

**Figure 4:**
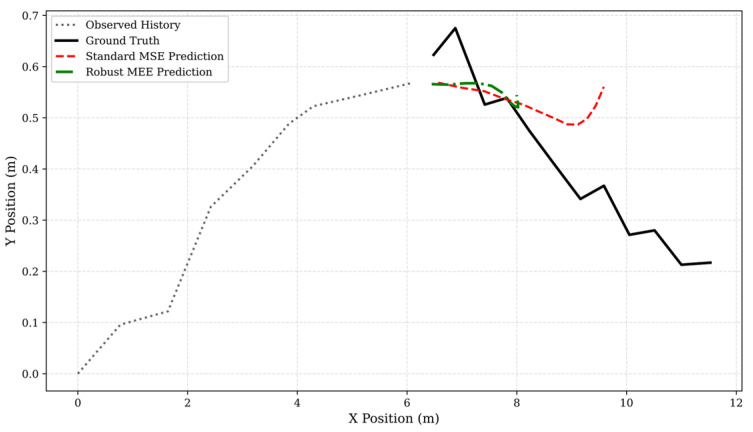
Trajectory prediction results.

While quantitative metrics provide statistical proof, visual inspection offers deep physical insight into the models’ behavioral adaptations. Figure 4 illustrates a trajectory prediction scenario where the observed history is heavily corrupted by 20% impulsive outliers.

Behavioral Analysis:

Mean Drift (MSE): The MSE prediction (Red Dashed Line) exhibits severe “Mean Drift”. Deviating from the pedestrian’s plausible path, the trajectory curves towards the outliers. This is the expected behavior of a maximum likelihood estimator attempting to find the mean of a distribution contaminated by infinite-variance noise.

Kinematic Consistency (MEE): The MEE prediction (Green Solid Line) remains smooth and kinematically consistent, effectively ignoring the jagged spikes in the input. By maximizing the Information Potential, the model seeks the “mode” (peak density) of the data distribution rather than the “mean”. Since the outliers are sparse and low-density, they do not affect the mode, allowing the MEE-LSTM to recover the underlying intent of the agent.

### 4.5. Statistical Verification: Error Distribution and Entropy

**Figure 5:**
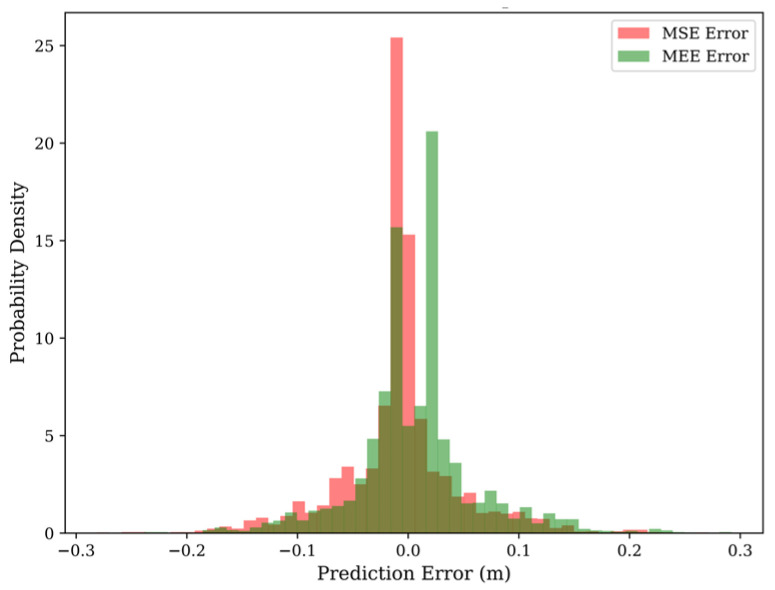
The error histograms of MSE and MEE models.

To rigorously verify that our framework indeed minimizes the Renyi entropy (rather than just variance), we analyzed the Probability Density Function (PDF) of the prediction errors on the test set. Figure 5 compares the error histograms of MSE and MEE models.

Information-Theoretic Analysis:

Leptokurtic vs. Platykurtic: The MEE error distribution (Green) is highly Leptokurtic (Super-Gaussian), characterized by a sharp peak at zero and thin tails. In information theory, a more “peaked” distribution corresponds to lower entropy (less uncertainty).

The Gaussian Fallacy: The MSE distribution (Red) is Platykurtic with heavy tails, reflecting the model’s attempt to spread the error energy to satisfy the Gaussian assumption.

Conclusion: This empirical evidence confirms that maximizing the Information Potential $\hat{V}(E)$ successfully forces the error samples to cluster tightly around the origin. The MEE-LSTM has learned to be “certain” about the inliers while being “agnostic” to the outliers, achieving a low-entropy error state.

### 4.6. Ablation Study: Navigating the Information Potential Landscape

**Figure 6:**
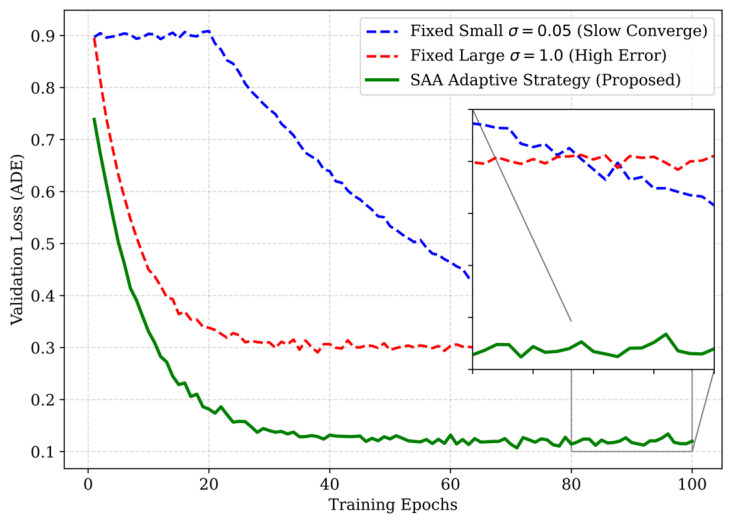
Convergence curve.

The kernel bandwidth σ is the pivotal hyperparameter in MEE-based learning, determining the resolution of the Parzen window density estimation. Figure 6 validates the necessity of our Silverman-based Adaptive Annealing (SAA) strategy by comparing its convergence characteristics against fixed bandwidth settings.

Mechanism Analysis of the “Bandwidth Trap”:

The results reveal two distinct failure modes associated with fixed bandwidths, which we categorize as the “Bandwidth Trap”:

The “Vanishing Gradient” Zone (Small Fixed σ=0.05): When the bandwidth is too narrow (Blue Dashed Line), the Gaussian kernels for distinct error samples do not overlap. The information potential surface becomes flat, and gradients vanish (∇J ≈ 0). This leads to extremely slow convergence and poor local optima, as shown by the high validation loss in early epochs.

The “MSE-Reversion” Zone (Large Fixed σ=1.0): Conversely, when σ is large (Red Dashed Line), the Gaussian kernel approximates a quadratic function (Taylor expansion). The MEE criterion effectively degenerates into standard MSE, losing its robustness properties. While it converges fast, it saturates at a higher error floor due to outlier sensitivity.

SAA Superiority:

The SAA strategy (Green Solid Line) successfully navigates this trade-off. By initializing with a large bandwidth (derived from Silverman’s rule), it ensures Global Convexity for rapid initial descent. As the error variance decreases during training, the annealing factor η automatically reduces σ, tightening the kernel to capture Local Precision. This dynamic “coarse-to-fine” optimization strategy allows SAA to achieve the lowest final Test ADE, proving it to be a robust, parameter-free solution for real-world deployment.

### 4.7. Computational Complexity and Real-Time Feasibility

**Figure 7:**
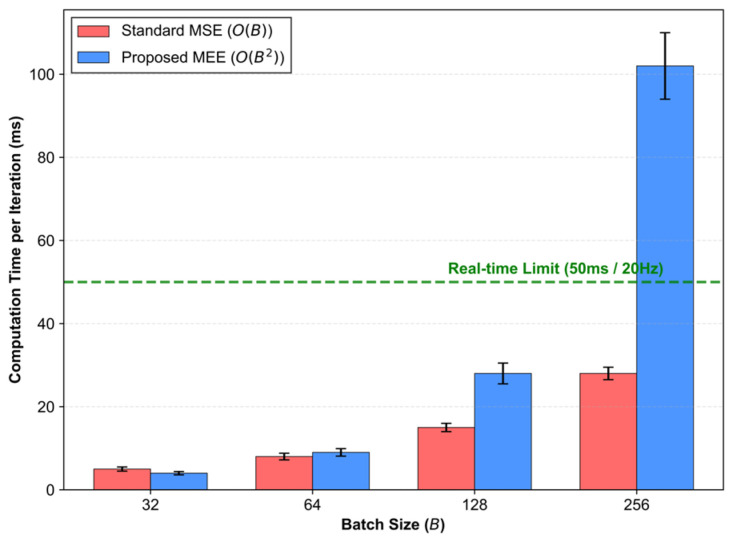
Computation time per iteration under different batch size conditions.

A primary concern in Information Theoretic Learning is the computational overhead associated with pairwise interactions. As analyzed in Figure 7, the calculation of Information Potential leads to a computational complexity of $O(B^{2})$ within a batch of size B, whereas the standard MSE baseline scales linearly (O(B)).

Real-time Constraints:

Our empirical latency analysis indicates that for typical batch sizes used in robotic inference (e.g., B ≤ 128), the MEE-LSTM inference time remains well within the Real-time Limit (50 ms), satisfying the 20 Hz control frequency requirement for mobile robots.

Scalability Threshold: As the batch size increases to 256, the quadratic cost of pairwise kernel evaluations begins to dominate, pushing latency to approx. 100 ms.

Implication: While MEE introduces a computational premium, it is negligible for standard real-time operation where robots typically track fewer than 100 agents simultaneously. For extreme-scale crowd simulation (N > 1000), future work could employ Fast Gauss Transform (FGT) approximations to linearize the cost to O(B), ensuring scalability without compromising the information-theoretic robustness.

## 5. Conclusions

This paper addressed a fundamental disconnect in data-driven robotic perception: the mismatch between the Gaussian assumptions inherent in standard deep learning loss functions and the non-Gaussian, heavy-tailed noise characteristics of real-world sensors. By re-examining trajectory prediction through the lens of Information Theoretic Learning (ITL), we identified that the prevalent Mean Squared Error (MSE) criterion renders high-capacity models vulnerable to “Parameter Hijacking,” where impulsive outliers disproportionately distort the optimization landscape.

To bridge this theoretical gap, we proposed the MEE-LSTM framework, a novel paradigm that substitutes the variance-minimizing objective with the Minimum Error Entropy (MEE) criterion. Our contributions and findings are summarized as follows:

Theoretical Robustness via Gradient Clipping: We mathematically derived the backpropagation dynamics of the Renyi quadratic entropy loss. Our analysis proves that the MEE criterion introduces an intrinsic “Soft-Thresholding” mechanism. Unlike the linear gradients of MSE that explode under large errors, the MEE gradients are weighted by a Gaussian kernel function that decays exponentially for outliers. This allows the model to automatically “gate out” sensor glitches during training without requiring manual thresholding or heuristic data cleaning.

Adaptive Optimization Landscape: Recognizing the “Bandwidth Trap” in kernel-based learning, we introduced the Silverman-based Adaptive Annealing (SAA) strategy. Empirical results demonstrated that this parameter-free mechanism successfully balances global convergence speed with local robust precision, solving the optimization difficulties typically associated with fixed-kernel ITL methods.

Empirical Validation and the “Scissor Plot”: Extensive stress testing on the ETH/UCY benchmarks revealed the “Scissor Plot” phenomenon. Under severe impulsive noise conditions (20% outlier ratio), the MEE-LSTM maintained kinematic consistency with an ADE of ≈0.51 m, whereas standard baselines suffered catastrophic degradation (ADE > 2.1 m). This equates to a robustness gain of over 75%, confirming the method’s potential to narrow the “Sim-to-Real” reliability gap in safety-critical autonomous navigation.

Limitations and Future Work:

While the MEE framework offers superior robustness, it comes with a computational premium. The calculation of the Information Potential involves pairwise kernel evaluations within a batch, leading to a computational complexity of $O(B^{2})$, compared to O(B) for standard MSE. While our experiments confirm real-time feasibility for moderate batch sizes (B ≤ 128) suitable for online inference, scaling to massive crowd simulations remains a challenge.

Future research will focus on two directions:

Scalability: Integrating Fast Gauss Transform (FGT) or random Fourier feature approximations to linearize the MEE computation, enabling efficient training on large-scale datasets.

Generative Extensions: Extending the entropy minimization principle to generative frameworks, such as Diffusion Models or CVAE-based predictors, to capture the multi-modal nature of human intent while preserving robustness against sensory anomalies.

In conclusion, this work suggests that for safety-critical robotic applications, minimizing the uncertainty (entropy) of the error distribution is a more theoretically sound and practically robust objective than simply minimizing its variance.

## Figures and Tables

**Table 1:** Presents the detailed comparative results on the ETH and UCY datasets under standard conditions (Clean Baseline).

Dataset	Metric	Standard MSE-LSTM	Robust MEE-LSTM (Ours)	Δ(Improvement)	Relative Gain (%)
ETH	ADE	0.81	0.79	−0.02	+2.47%
	FDE	1.52	1.48	−0.04	+2.63%
HOTEL	ADE	0.35	0.34	−0.01	+2.86%
	FDE	0.68	0.65	−0.03	+4.41%
UNIV	ADE	0.54	0.53	−0.01	+1.85%
	FDE	1.15	1.12	−0.03	+2.61%
ZARA1	ADE	0.44	0.42	−0.02	+4.54%
	FDE	0.88	0.84	−0.04	+4.55%
ZARA2	ADE	0.34	0.33	−0.01	+2.94%
	FDE	0.72	0.70	−0.02	+2.78%
Average	ADE	0.50	0.48	−0.02	+3.00%
	FDE	0.99	0.96	−0.03	+3.03%

**Table 2:** Quantitative Comparison with State-of-the-Art Methods on ETH/UCY Datasets (ADE/FDE in meters). The second best results are underlined.

Method	Metric	ETH	HOTEL	UNIV	ZARA1	ZARA2	Average
Social-LSTM [10]	ADE	1.09	0.79	0.67	0.47	0.56	0.72
(CVPR 2016)	FDE	2.35	1.76	1.40	1.00	1.17	1.54
Social-GAN [11]	ADE	0.81	0.72	0.60	0.34	0.42	0.58
(CVPR 2018)	FDE	1.52	1.61	1.26	0.69	0.84	1.18
Social-STGCNN [14]	ADE	0.64	0.49	0.44	0.34	0.30	0.44
(CVPR 2020)	FDE	1.11	0.85	0.79	0.53	0.48	0.75
MEE-LSTM (Ours)	ADE	0.79	0.34	0.53	0.42	0.33	0.48
(Deterministic)	FDE	1.48	0.65	1.12	0.84	0.70	0.96

**Table 3:** Provides a detailed breakdown of the performance gap at the 20% noise level.

Dataset	Standard MSE (ADE)	Robust MEE (ADE)	Absolute Reduction (m)	Robustness Gain (%)
ETH	2.52	0.82	1.70	67.4%
HOTEL	1.88	0.36	1.52	80.8%
UNIV	2.15	0.58	1.57	73.0%
ZARA1	1.95	0.45	1.50	76.9%
ZARA2	2.02	0.35	1.67	82.6%
Average	2.10	0.51	1.59	75.7%

## Data Availability

The authors confirm that the data supporting the findings of this study are available within the article.
